# Circulating microRNAs in cancer: origin, function and application

**DOI:** 10.1186/1756-9966-31-38

**Published:** 2012-04-30

**Authors:** Ruimin Ma, Tao Jiang, Xixiong Kang

**Affiliations:** 1 Laboratory Diagnosis Center, Beijing Tian Tan Hospital, Capital Medical University, No.6 Tiantan Xili, Dongcheng District, Beijing, 100050, China; 2 Department of Neurosurgery, Beijing Tian Tan Hospital, Capital Medical University, No.6 Tiantan Xili, Dongcheng District, Beijing, 100050, China

**Keywords:** MicroRNA, Circulating, Cancer, Diagnosis

## Abstract

MicroRNAs (miRNAs) are a class of small non-coding RNAs that regulate gene expression at the posttranscriptional level. The dysregulation of miRNAs has been linked to a series of diseases, including various types of cancer. Since their discovery in the circulation of cancer patients, there has been a steady increase in the study of circulating miRNAs as stable, non-invasive biomarkers. However, the origin and function of circulating miRNAs has not been systematically elucidated. In this review, we summarize the discovery of circulating miRNAs and their potential as biomarkers. We further emphasize their possible origin and function. Finally, we discuss the application and existing questions surrounding circulating miRNAs in cancer diagnostics. Although several challenges remain to be concerned, circulating miRNAs could be useful, non-invasive biomarkers for cancer diagnosis.

## Introduction

MicroRNAs (miRNAs) are approximately 22 nucleotides long, endogenous, single-stranded, non-protein-coding RNA molecules that regulate gene expression at the posttranscriptional level. Since their discovery in 1993, miRNAs have caused worldwide interest due to their characteristic function and modes of action, providing a new understanding of the central dogma of molecular biology. MiRNAs have been shown to regulate a variety of cellular processes, such as proliferation, differentiation, metabolism, ageing and cell death. As such, the importance of miRNAs is increasingly recognized in almost all fields of biological and biomedical fields [1]. In humans, it has been estimated that there are more than 1000 miRNAs in the genome which regulate approximately 60% of all protein-coding genes [2,3].

Recently, the importance of miRNAs in oncogenesis has been recognized. Dysregulation of miRNA expression plays a key role in cancer development through various mechanisms including deletions, amplifications, epigenetic silencing, or mutations in miRNA loci, the dysregulation of transcription factors that target specific miRNAs [4]. MiRNAs expression profiling studies, using microarrays and other methods, can be used to differentiate normal from cancer tissues as well as to classify different tumor types and grades. Furthermore, specific miRNAs expression features have been found to correlate with cancer prognosis and therefore have the potential to be used to determine the course of treatment [5-7].

The discovery of circulating miRNAs in cancer patients holds great promise for the use of miRNAs as distinctive, non-invasive cancer biomarkers. In this review, we focus on the origin and function of circulating miRNAs, and discuss their characteristics and their potential application as powerful biomarkers in cancer diagnostics. We also discuss some issues that need to be further explored and normalized before miRNAs can be successfully applied as biomarkers in a clinical setting.

## Biomarkers in the circulation

Circulating biomarkers undoubtedly play an increasingly significant role in clinical applications such as disease diagnostics, monitoring therapeutic effect and predicting recurrence in cancer patients. The currently used fluid-based biomarkers are primarily proteins, such as alpha-fetoprotein (AFP) [8], chromogranin A (CgA) [9], nuclear matrix protein 22 (NMP 22) [10], carbohydrate antigen 125 (CA 125) [11]; enzymes, such as prostate specific antigen (PSA) [12]; and human chorionic gonadotropin (hCG) [13]. While these biomarkers provide an opportunity to analyze tumors comprehensively in an invasive way, low sensitivity and specificity limit their clinical application. For example, serum levels of AFP are often elevated in hepatocellular carcinoma (HCC); however, this is also the case in germ cell tumors, gastric, biliary and pancreatic cancers. Moreover, serum levels of AFP are not consistently elevated in HCC patients, but are commonly found at normal or decreased levels [14]. Even for PSA, which is considered a sensitive biomarker for advanced prostate cancer, serum levels are often increased in men with benign prostatic hyperplasia [15]. These points underscore the importance of finding novel circulating biomarkers, such as miRNAs, to supplement biomarkers currently used in tumor classification and prognostication.

Chim et al. first identified the expression of miRNAs in the circulation in 2008. They used quantitative reverse-transcription polymerase chain reaction (qRT-PCR) to quantify miRNAs levels of apparent placental origin, in the plasma of pregnant women [16]. Shortly thereafter, Lawrie et al. reported elevated serum levels of miR-155, miR-210, miR-21 in diffuse large B-cell lymphoma patients compared with healthy controls. Moreover, high miR-21 expression was correlated to relapse-free survival [17]. These studies opened up the exciting prospect of utilizing circulating miRNAs as powerful, non-invasive diagnostic markers for cancers and other diseases.

Circulating miRNAs have many of the essential characteristics of good biomarkers. First, they are stable in the circulation and resistant to storage handling. Serum miRNAs are resistant to RNase digestion and other harsh conditions such as extreme pH, boiling, extended storage, and multiple freeze-thaw cycles. Second, most miRNAs sequences are conserved across species. Third, in some cases, changes in miRNA levels in circulation have been associated with different diseases as well as certain biological or pathological stages. Finally, miRNAs levels can easily be determined by various methods [18-23].

Several major profiling platforms are used today in miRNAs detection. A powerful method for the analysis of serum miRNAs involves relative quantification by stem-loop RT-PCR. This method has been widely used for the sensitive detection of low abundance circulating miRNAs [24]. Microarray analysis is also commonly used in miRNAs detection but generally requires more starting material than qRT-PCR, and can be complicated by the need to develop probes and hybridization conditions that can detect a variety of miRNAs concurrently [18]. Deep sequencing appears to be a very promising technique for identifying novel miRNA biomarkers [25]. This technology can be used to identify tissue and stage specific expression, and compare data with miRNAs profiles in different diseases [26-28]. These methods open exciting avenues for non-invasive quantification of miRNAs. However, reproducibility among different methods remains a major concern. Chen et al. found a weak correlation between results obtained by qRT-PCR array and oligonucleotide microchip methods, indicating considerable variability between the two assay platforms [29]. Clearly, more work is necessary to identify suitably standardized and normalized protocols.

## Origin of circulating miRNAs

The question of whether tumor-associated miRNAs detected in circulation results from tumor cell death and lyses, or instead from secretion by tumor cells remains unanswered. The latest findings concerning exosomal miRNAs could uncover the miRNA secretory mechanism.

As previously mentioned, miRNAs have proven to be robust against external factors, such as enzymatic degradation, freeze-thaw cycles, and extreme pH conditions [30,31]. Mitchell et al., by applying multiple steps of filtration and centrifugation to separate cells from plasma and recover RNA from both sections, demonstrated that serum miRNAs were not associated with cells or larger cell fragments, but existed in a stable and protected form [30]. The unexpected stability of circulating miRNAs in blood begs the question of what mechanism protects circulating miRNAs from degradation. Recent studies have revealed that miRNAs may be protected either in microvesicles (up to 1 μm) or in small membrane vesicles of endocytic origin called exosomes (50–100 nm) [32,33]. Kosaka and colleagues found that miRNA are first incorporated into exosomal particles, after which a surge of cellular ceramide stimulates the release of exosomes. Ceramide biosynthesis is regulated by neutral sphingomyelinase (nSMase). Treated HEK293 cells with nSMase inhibitor, GW4869, extracellular endogenous miR-16 and miR-146a were reduced in a dose-dependent manner, while their cellular expression levels remained unchanged. Furthermore, miRNAs packaged in exosomes can be delivered to recipient cells where they exert gene silencing through the same mechanism as cellular miRNAs [34]. Another study by Pigati suggests that miRNAs release into blood, milk and ductal fluids is selective and that this selectivity may correlate with malignancy. In particular, while the bulk of miR-451 and miR-1246 produced by malignant mammary epithelial cells were released, the majority of these miRNAs produced by non-malignant mammary epithelial cells was retained [35]. It therefore seems likely that the profiling of secretory miRNAs could be a valuable cancer diagnostic and prognostic tool. However, intercellular trafficking mechanism that determines whether miRNAs are secreted or retained in their originating cells requires further investigation [36].

While secretory miRNAs have been hypothesized to be involved in mediating cell-cell communication, it remains unclear whether all extracellular miRNAs are associated with exosomes. Different opinions exist regarding this issue. Using a mammalian cell culture model, Wang et al. [37] showed that a significant fraction of extracellular miRNAs resided outside of vesicles and acted in exosome-independent manner. A number of RNA-binding proteins, most importantly nucleophosmin 1 (NPM1), which were released into the cell culture medium together with miRNAs may play a role in protecting miRNAs from degradation. Another study by Turchinovich et al. [38] found that most miRNAs in plasma and cell culture media completely passed through 0.22 μm filters but remained in the supernatant after ultracentrifugation at 110000 × g, indicating a non-vesicular origin of extracellular miRNAs. In addition to revealing that extracellular miRNAs were predominantly free of exosomes or microvesicles, they demonstrated an association between miRNAs and the argonaute protein Ago2, an RNA-induced silencing complex-related protein. They hypothesized that circulating miRNAs were mostly by-products of dead/dying cells that remain stably complexed to Ago2 in the extracellular environment. However, some miRNA/Ago2 complexes may be actively released from cells and act in a paracrine manner. Furthermore, the authors of this study do not reject the possibility that some miRNAs may be associated with exosomes.

A third possibility exists. A large proportion of circulating miRNAs are likely derived from blood cells and other organs it is therefore possible that cancer-associated miRNAs in the circulation may originate from immunocytes in the tumor microenvironment or from some other response mediated by the affected organ or system. Tumor cells secrete a variety of miRNAs that act on immunocytes to modulate immune responses and create either an immunostimulatory or an immunotolerant tumor environment. Conversely, immunocytes may secrete cancer-associated miRNAs, thereby promoting or inhibiting proliferation, invasion and apoptosis. As an example, there is an inverse correlation between miR-17-92 expression and transforming growth factor-β receptor II (TGFBR2) transcript levels in CD 34^+^ hematopoietic stem cells [39]. Furthermore, TGFBR2 is a verified target of miR-17-92 in solid cancers [40]. It is therefore hypothesized that miR-17-92, expressed in T cells, down-regulates TGFBR2 expression, thereby making T cells more resistant to the immunosuppressive effects of TGF-β, which is often expressed at high levels in glioma [41]. Another miRNA, miR-21, was reported to control inflammation by regulating the pro-inflammatory tumor suppressor programmed cell death 4 (PDCD4) thereby promoting IL-10 production in macrophages [42]. On the other hand, miR-21 was found to promote tumorigenesisi by downregulating phosphatase and tensin homologue (PTEN) and activating v-akt murine thymoma viral oncogene homolog (AKT) [43]. One of the first miRNAs linked with cancer, miR-155, upregulated by inflammatory stimuli in macrophages [44]. These links between alterations in miRNAs levels in inflammatory reaction and tumorigenesis indicate that cancer-associated miRNAs in the circulation may originate from the immunologic system, and that dysregulation of miRNAs may be an important link between immunity and cancer.

Identifying the relationship between circulating miRNAs and tissue miRNAs will be helpful in understanding the origin of circulating miRNAs. Most studies to date found the same trend of alteration between circulating miRNAs and tissue miRNAs. For instance, Brase et al. found that miR-375 and miR-141 were both highly expressed in serum and tissue samples of prostate cancer patients [45]. The levels of five miRNAs (miR-17-3p, miR-135b, miR-222, miR-92 and miR-95) were also found to be elevated in plasma and tissue samples of colorectal cancer patients [46]. However, Wulfken et al. found that 109 miRNAs were at higher levels in renal cell carcinoma patients’ serum, but only 36 miRNAs were upregulated in the corresponding tissue samples. It is possible that only a subset of circulating miRNAs have tumor-specific origins [47]. Another study reported that about 66% but not all of the released miRNAs reflects the cellular miRNAs abundance of malignant mammary epithelial cells. These data suggest that cells have a mechanism in place to select specific miRNAs for cellular release or retention [35]. These studies therefore demonstrate different sources of circulating miRNAs, which makes it possible for circulating miRNAs to reflect every aspect of the human physiological state.

## Circulating miRNAs function

It is estimated that miRNAs regulate approximately 60% of all protein-coding genes. Mature miRNAs regulate gene expression by binding to complementary sites in the target mRNA. The degree of complementarity between miRNAs and their targets seems to determine the regulating results [48]. MiRNAs that bind to protein-coding mRNA sequences with perfect complementarity could induce the RNA-mediated interference (RNAi) pathway, leading to cleavage of mRNA by Ago2 in the RNA-induced silencing complex (RISC) [49]. However, imperfect base pairing between miRNA and the target mRNA exists much more frequently in mammals. In this case, miRNAs act by binding to sites within the 3′ untranslated regions (3′UTRs) of their target protein-coding mRNAs, leading to inhibition of expression of these genes at the level of translation [50,51].

Recently, some studies have identified a number of miRNAs that activate the expression of certain target genes in a sequence-specific manner instead of silencing them [1]. Place et al. found that miR-373 induced expression of E-cadherin and cold-shock domain-containing protein C2 (CSDC2) genes with complementary sequences in their promoters [52]. This novel mechanism is named “RNA activation” (RNAa), a process that may require the Ago2 protein and could be associated with histone changes linked to gene activation [53]. The discovery of RNAa introduces a new understanding of miRNA function which, in addition to an inhibitory effect, miRNAs may also promote expression in certain instances.

Regarding their effect on cell biology, miRNAs can have a profound effect on tumorigenesis. There is evidence for a range of the modulatory effects of miRNAs including cell proliferation, angiogenesis, apoptosis, metastasis, invasion, and other biological processes. For instance, miR-17-92 cluster can promote proliferation, increase angiogenesis, and sustain cancer cell survival via post-transcriptional repression of target mRNAs [54]. The let-7 family, which were down-regulated in many malignancies, inhibited cancer growth by targeting key regulators of mitogenic pathways, such as RAS and high mobility group A2 (HMGA2) [55]. miR-10b was highly expressed in metastatic breast cancer cells and positively regulated cell migration and invasion. Its overexpression in otherwise non-metastatic breast tumors also initiated robust invasion and metastasis [56]. miR-373 stimulated breast tumor cell migration and invasion by suppressing CD44 gene expression [57]. As another example, miR-125b was found to inhibit apoptosis in neuroblastoma cells in a p53-dependent manner [58]. Taken together, these studies indicate that miRNAs have crucial effects in carcinogenesis and can either act as oncogenes or tumor-suppressor genes.

**Figure 1:**
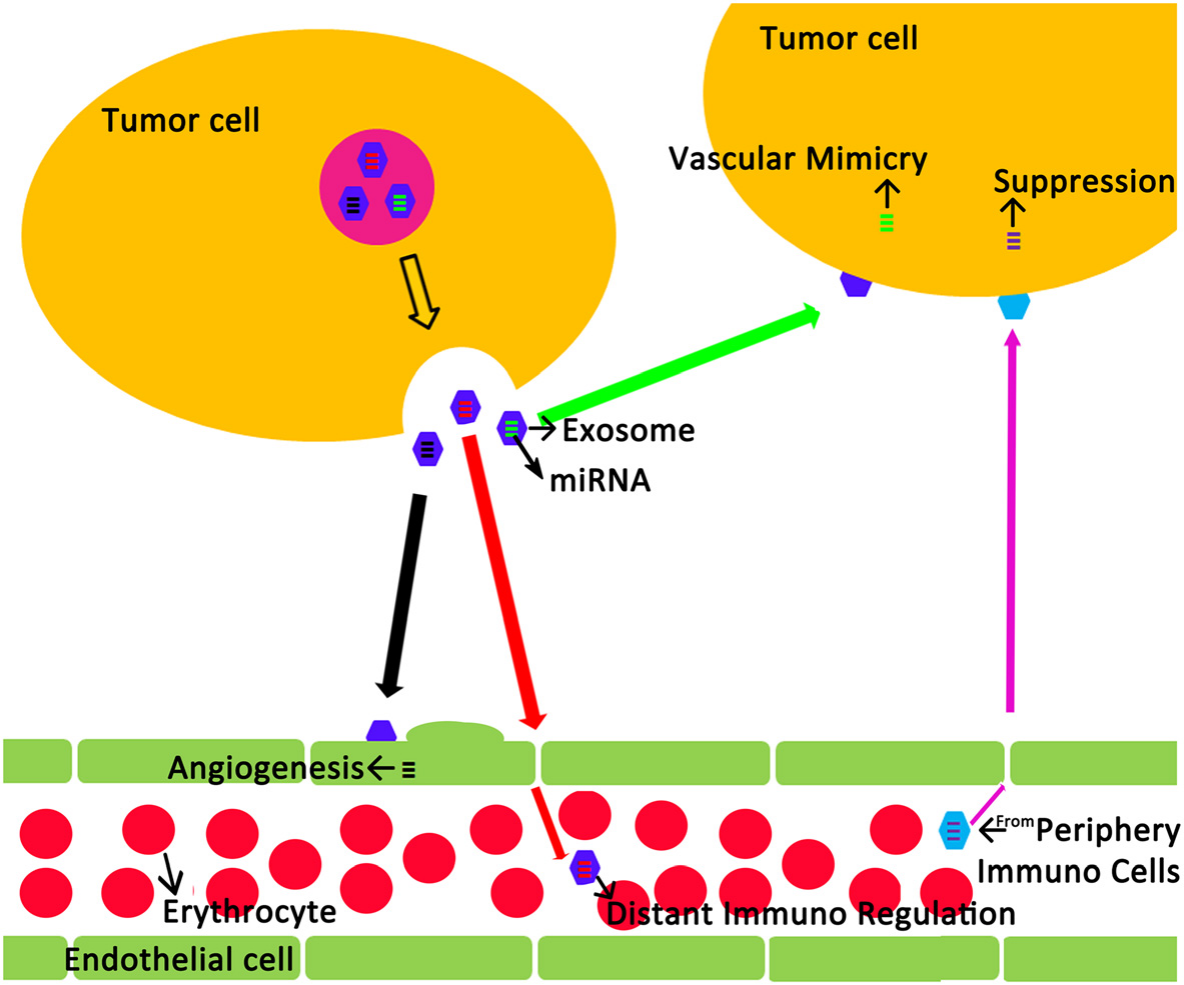
**Functional pattern of circulating miRNAs in cancer cells.** Circulating miRNAs released by tumor cells may involve in angiogenesis, distant immunoregulation and vascular mimicry of tumor cells. Periphery immunocytes may secrete tumor-suppressive miRNAs to block tumor growth and propagation.

Circulating miRNAs may have specific roles that are dependent on their origin (Figure 1). Cancer cells may evade the attacks of T and B cells by releasing immunosuppressive miRNAs. Cancer cells may also recruit capillary blood vessels with angiogenic miRNAs. Alternatively, surrounding cells may secrete tumor- suppressive miRNAs, which block tumor growth and propagation. Once the balance is disrupted, expansive growth of cancer cells may follow [59-61]. Microvesicles derived from human melanomas and colorectal carcinomas promote tumor growth and immune escape by skewing monocyte differentiation towards TGF β-secreting myeloid suppressive cells [62]. On the other hand, miRNA-containing exosomes, produced by dendritic cells and B lymphocytes, can deliver the optimal signal for T cell activation. However, in some instances they can also maintain peripheral tolerance by inducing anergy in specific T cells or activation-induced cell death, depending on the functional status of the originating cells. MiRNAs released from tumor cells and immunocytes may therefore work together resulting in poor clinical outcomes [63-65].

MiRNAs are important modulators of tumor-associated angiogenesis. The miR-17-92 cluster, which includes miR-17, miR-18a, miR-19a/b, miR-20a, and miR-92a, has been linked to tumor angiogenesis. Overexpression of the entire miR-17-92 cluster in myc-induced tumors has been found to increase angiogenesis by paracrine signaling [66]. However, overexpression of the individual members of the miR-17-92 cluster reduced endothelial cell sprouting, while inhibitors of these miRNAs augmented angiogenesis in vitro, indicating that the miR-17-92 cluster provides a cell-intrinsic antiangiogenic activity in endothelial cells [67]. Another study by Grange et al. [68] found that microvesicles released from CD105^+^ renal cancer stem cells, in which 57 miRNAs were differentially expressed, contributed to triggering the angiogenic switch and coordinating metastatic diffusion during tumor progression. While miR-27b and let-7f were described as proangiogenic miRNAs, miR-221 and miR-222 were identified as antiangiogenic miRNAs in endothelial cells [69-71]. MiRNAs may also influence angiogenesis by acting on endothelial progenitor cells (EPCs) since EPCs play an important role in neovascularization. miR-34a was reported as a tumor suppressor and regulates cell cycle, senescence, apoptosis, and metabolism [72,73]. A recent study found that overexpression of miR-34a in EPCs impaired EPC-mediated angiogenesis by inducing senescence via the inhibition of silent information regulator 1 (SIRT 1). This study provided a mechanistic insight on miRNA-mediated regulation of EPC function [74]. The question of whether in the course of EPC homing to tumor cells, circulating miRNAs have some specific function remains unanswered. They could conceivably act as chemokines, which direct EPCs to tumor neovessels and promote vessel growth [75]. This topic certainly warrants further investigation.

## Application of circulating miRNAs

Their stability and predictive property make miRNAs ideal serum and plasma biomarkers in cancer patients. A variety of independent studies have successfully proved the importance of miRNAs as a tool of cancer diagnosis. Wu and colleagues found that miR-21and miR-29 were significantly upregulated in the serum of breast cancer patients and may be useful biomarkers for breast cancer detection [76,77]. In non-small cell lung cancer (NSCLC), the expressions of miR-1254 and miR-574-5p were significantly increased with respect to controls. They were able to discriminate tumor samples from controls with 82% and 77% sensitivity and specificity, respectively, as judged by the use of a receiver operating characteristic (ROC) curve [78]. Wei et al. also reported that plasma levels of miR-21 were significantly higher in NSCLC patients compared with their age- and sex-matched controls, and that miR-21 can serve as a circulating tumor biomarker for the early diagnosis of NSCLC [79]. Other studies provide further support for the use of circulating miRNAs as non-invasive biomarkers for a wide range of cancers, including hepatocellular carcinoma [80,81], malignant melanoma [82] and gastric cancer [83] (Table 1). Moreover, researchers found that circulating miRNAs might be used to detect early stage cancer. Zheng et al. reported that the levels of miR-155, miR-197 and miR-182 in the plasma of lung cancer patients, including stage I cancers, were significantly elevated compared with controls. The combination of these three miRNAs yielded 81.33% sensitivity and 86.76% specificity in discriminating lung cancer patients from controls [84]. Schrauder and colleagues performed microarray-based miRNA profiling on whole blood from 48 breast cancer patients at diagnosis along with 57 healthy individuals as controls. All breast cancers were histologically confirmed as early stage invasive ductal carcinoma of the breast with a tumor size ranging between 0.15 and 4.0 cm. They found that 59 miRNAs were significantly differentially expressed in whole blood from cancer patients compared with healthy controls, and that 13 and 46 miRNAs were significantly up- or down-regulated, respectively [85]. Bianchi et al. developed a test, based on the detection of 34 miRNAs from serum, that could identify early stage NSCLC in a population of asymptomatic high-risk individuals with 80% accuracy [86].

**Table 1:** Circulating miRNAs as diagnostic markers for different human cancers

**Disease**	**miRNA**	**Expression level**	**Contributors**
**Breast cancer**	miR-29a	Up-regulation	**Wu et al.**, J Biomed Biotechnol. (2010) [76]
	miR-21		**Asaga et al.**, Clin Chem. (2011) [77]
**Lung cancer**	miR-21,1254,574-5p	Up-regulation	**Wei et al.**, Chin J Cancer. (2011) [79]
			**Foss et al.**, J Thorac Oncol. (2011) [78]
**Hepatocellular carcinoma**	miR-16,miR-199a	Down-regulation	**Qu et al.**, J Clin Gastroenterol. (2011) [80]
	miR-21,miR-122,miR-223	Up-regulation	**Xu et al.**, Mol Carcinog. (2010) [81]
**Malignant melanoma**	miR-221	Up-regulation	**Kanemaru et al.**, J Dermatol Sci. (2011) [82]
**Gastric cancer**	miR-1,20a,27a,34,423-5p	Up-regulation	**Liu et al.**, Eur J Cancer. (2011) [83]

In addition, some miRNAs may be useful prognostic biomarkers for different cancers. Hu et al. [87] used Solexa sequencing followed by qRT-PCR to test the difference in serum levels of miRNAs between NSCLC patients with longer and shorter survival. Eleven serum miRNAs were found to be altered more than five-fold between the two groups. Levels of four miRNAs (miR-486, miR-30d, miR-1 and miR-499) were significantly associated with overall survival, and this four-miRNA signature may serve as a predictor for overall survival in NSCLC patients. Cheng et al. [88] found that plasma miR-141 was an independent prognostic factor for advanced colon cancer and that high plasma levels of miR-141 were associated with poor prognosis. Another study identified miR-221 as a significant prognostic factor for poor overall survival in colorectal cancer patients [89]. Additionally, researchers reported the usefulness of circulating miRNAs in evaluating treatment-response in cancer patients. For instance, serum levels of miR-21 levels were elevated in hormone-refractory prostate cancer patients, especially in those resistant to docetaxel-based chemotherapy, making miR-21 a potential predictor for the efficacy of docetaxel-based chemotherapy [90]. These findings demonstrate that circulating miRNAs may be useful in predicting patterns of sensitivity and resistance to anti-cancer drugs.

Since the application of circulating miRNAs to the field of cancer diagnostic is still new, certain points remain to be explored and normalized. One important issue that needs to be addressed is the suitability of different sample types for miRNA detection. While Mitchell et al. found no significant differences when comparing serum and plasma levels of miRNAs [30,91], this result was limited to only four miRNAs and might not reflect the global situation. Recently, researchers found that serum samples yielded lower miRNA concentrations [92]. Further study indicated that the higher concentrations of miRNAs in plasma compared with serum were mainly due to the presence of cellular contaminants, and in particular, platelets. To minimize the variation introduced by variable levels of platelet contamination, serum samples should be more suitable. Meanwhile, centrifugation protocols used to separate serum or plasma require normalization before results can be compared [93].

Another crucial issue is the use of appropriate normalization controls. So far, several normalization strategies have been used for the analysis of circulating miRNAs. There is however no consensus. Some genes such as RNU6B, 18S rRNA or 5S rRNA have been used to normalize data [94,95], but other researchers considered them highly variable or sensitive to degradation [96]. miR-16 has been used in many studies as an internal normalization control [97,98], but was later found to be susceptible to hemolysis and was related to some diseases that would make it unstable in circulation [80,93,99]. Synthetic *C*. *elegans* miRNAs, such as Cel-miR-39 and Cel-miR-54 have been used as spike-in controls during RNA isolation [100,101]. However, they were later found to be degraded by RNase in the circulation. For the above reasons, some researchers chose to perform normalization without the use of a reference gene. For instance, Hu et al. [87] used a healthy donor sample, which was processed together with the test samples, to control for technical variability. Since the coefficient of variation (CV) of Ct values for the control sample between different plates for different miRNAs was small, test reactions were comparable between different plates. In addition, both the volume of serum and eluent extracted for qRT-PCR were consistent throughout the study. This strategy may minimize the variances among samples and make the results more comparable [93]. Other issues that need to be addressed include poor correlation between different measurement platforms, lack of standardized protocols for sample preparation and a suitable method for measuring the concentration of miRNA in the circulation.

## Conclusions

The discovery of circulating miRNAs brought forward a new understanding of the basic mechanisms of oncogenesis and opened up exciting prospects for diagnostics and prognostics. Although still a new field, with much to be explored, the hope is to apply circulating miRNAs to cancer diagnosis and treatment, once we know more about their origin and function. However, before novel biomarkers can be routinely used in a clinical setting, standardized procedures for sample preparation as well as a proper method for normalization during analysis is essential. Large scale and independent clinical studies will also be required.

## Abbreviations

MiRNAs = MicroRNAs; qRT-PCR = quantitative reverse-transcription polymerase chain reaction; NPM1 = Nucleophosmin 1; TGF-β = Transforming growth factor-β; PDCD4 = Programmed cell death 4; PTEN = Phosphatase and tensin homologue; RNAi = RNA-mediated interference; RISC = RNA induced silencing complex; 3′UTRs = 3′ untranslated regions; CSDC2 = Cold-shock domain-containing protein C2; RNAa = RNA activation; EPCs = Endothelial progenitor cells.

## Competing interests

The authors declare that they have no competing interests.

## Authors’ contributions

Xixiong Kang initiated the concept. Ruimin Ma and Tao Jiang drafted the manuscript. All authors participated in writing, reading and approving the final manuscript.

## Authors’ information

Ruimin Ma: Laboratory Diagnosis Center, Beijing Tian Tan Hospital, Capital Medical University, No.6 Tiantan Xili, Dongcheng District, Beijing 100050, China

Tao Jiang: Department of Neurosurgery, Beijing Tian Tan Hospital, Capital Medical University, No.6 Tiantan Xili, Dongcheng District, Beijing 100050, China

Xixiong Kang: Laboratory Diagnosis Center, Beijing Tian Tan Hospital, Capital Medical University, No.6 Tiantan Xili, Dongcheng District, Beijing 100050, China
